# Cyclosporiasis Outbreak in Germany Associated with the Consumption of Salad

**DOI:** 10.3201/eid0809.10.3201/eid0809.010517

**Published:** 2002-09

**Authors:** Peter C. Döller, Karl Dietrich, Norbert Filipp, Stefan Brockmann, Caroline Dreweck, Reinhard Vonthein, Christiane Wagner-Wiening, Albrecht Wiedenmann

**Affiliations:** * Paul-Lechler-Clinic for Tropical Diseases, Tübingen, Germany; † Public Health Office, Reutlingen, Germany; ‡ State Health Office of Baden-Württemberg, Stuttgart, Germany; § Tübingen University Hospital, Tübingen, Germany; ¶ Eberhard-Karls-University, Tübingen, Germany

**Keywords:** food parasitology, *Cyclospora cayetanensis*, disease outbreak, gastroenteritis, lettuce, Europe

## Abstract

This outbreak is the first foodborne cyclosporiasis outbreak reported from central Europe. The illness was reported in 34 persons who attended luncheons at a German restaurant. The overall attack rate was 85% (34/40). The only foods associated with significant disease risk were two salad side dishes prepared from lettuce imported from southern Europe and spiced with fresh green leafy herbs (p=0.0025).

*Cyclospora cayetanensis*, a protozoan parasite, which was named and classified by Ortega et al. in 1994 (1), is endemic in geographic regions with warm or tropical climates (2,3). Cyclosporiasis typically has onset after an incubation period of approximately 1 week and is characterized by protracted and often relapsing gastroenteritis. Treatment is with trimethoprim-sulfamethoxazole (4). After two nationwide outbreaks of cyclosporiasis linked to raspberries imported from Guatemala (5) occurred in the United States and Canada in 1996–97, reports speculated that imported food could also cause outbreaks or sporadic infections in other regions with a temperate climate, such as central Europe (6,7).

## The Study`

**Figure 1:**
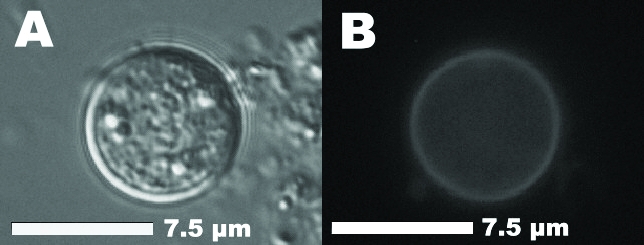
Unsporulated oocyst of *Cyclospora cayetanensis* in an unstained stool preparation. A) Differential interference contrast. B) Same oocyst with typical blue autofluorescence (Filter sets: 365-nm excitation, 395-nm dichroic mirror, 420-nm suppression).

On December 13 and 14, 2000, four independent parties of 6, 7, 7, and 20 persons attended holiday luncheons in a restaurant in southwest Germany. From December 29, 2000, to January 18, 2001, some of these persons contacted local health authorities because of protracted, sometimes relapsing gastroenteritis symptoms. After several stool specimens were negative for routine bacteriologic, virologic, and parasitologic tests, the patients who were still having gastrointestinal symptoms were referred to the outpatient department of a tropical medicine clinic for an examination for intestinal protozoa. *C. cayetanensis* was detected with a modified Ziehl-Neelsen technique in stool smears of 9 of 19 persons (8 attendees of the luncheons and the owner of the restaurant). The first laboratory-confirmed diagnosis was made 27 days after the peak of the outbreak, when the number of excreted oocysts still detectable in the stool smears was moderate or low. Confirmatory tests (epifluorescence microscopy, differential interference contrast, and object measurement with an electronic image analysis system) were performed at the State Health Office in Stuttgart and the Institute for General and Environmental Hygiene of the University of Tübingen (Figure 1).

All 40 attendees of the luncheons were asked to complete a questionnaire that included questions about age, gender, travel history, food items and beverages consumed at the luncheons, onset and duration of symptoms, physician consultation, examination of stool samples, antibiotic treatment, and days absent from work.

Using the criteria established by Herwaldt et al. (5), we defined cases of clinical cyclosporiasis as illness in persons who began to have at least one gastrointestinal symptom (diarrhea, flatulence, weight loss, nausea, abdominal cramps, or vomiting) 12 hours to 14 days after the event. Patients in whom typical oocysts were detected in at least one stool sample were defined as having laboratory-confirmed cases.

The statistical analysis was performed with the software packages Epi Info Version 6.04 (Centers for Disease Control and Prevention, Atlanta, GA) and StatXact 3.0.2 (Cytel Statistical Software, Cambridge, MA). Univariate relative risks with exact 95% confidence intervals and two-tailed p values of the unconditional test were calculated according to the procedure described in the manual of StatXact 3.0.2. (8).

According to initial reports from the four groups who had attended the luncheons, the overall attack rate was 85% (34 of 40 persons). Thirty of these 40 persons participated in the retrospective cohort study. Twenty-six persons had clinical cases; eight had laboratory-confirmed cases; and four did not become ill. The attack rate in the study participants was 87% (26 of 30 persons), i. e., the ratio of ill to non-ill persons in the retrospective cohort study was not substantially distorted. All participants were adults 22–65 years of age; 12 persons were men.

**Figure 2:**
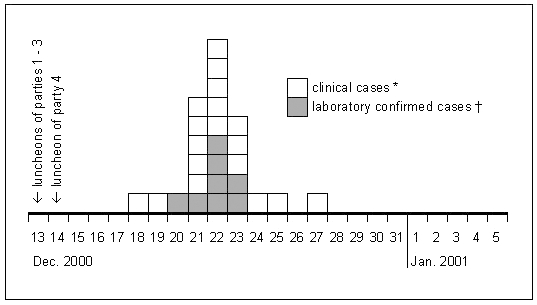
Epidemic curve of an outbreak of cyclosporiasis occurring in attendees of four holiday luncheons in a restaurant in Germany in December 2000. Results for the 26 cases in a retrospective cohort study. *Defined as having reported any of these symptoms: diarrhea (>3 bowel movements per day), loss of appetite, weight loss, flatulence, abdominal cramps, nausea, vomiting. †Laboratory confirmation by detection of *Cyclospora* oocysts in at least one stool sample by a modified Ziehl-Neelsen technique.

The epidemic curve of the outbreak is illustrated in Figure 2. The median incubation time was 8 days (range 5–14 days). Symptoms occurred with the frequencies listed in the table. The median duration of symptoms was 25 days (range 15–42 days; n=19). The outbreak caused a total of 80 days off work (range 2–24 days; n=12). Only four patients who consulted a physician before January 18, the day the first case was laboratory confirmed, had already received antibiotic treatment. At least one of them received a drug (amoxicillin) without documented effectiveness against *Cyclospora*. All patients with a laboratory-confirmed diagnosis of cyclosporiasis received a 7-day treatment of trimethoprim-sulfamethoxazole, starting immediately after diagnosis had been made.

**Table:** Symptoms associated with 26 cases of cyclosporiasis during a foodborne outbreak, Germany, December 2000

Symptom	No. with symptom / no. with available data (%)^a^
Diarrhea^b^	24/25 (96.0)
watery	23/24 (95.8)
mucus	11/18 (61.1)
bloody	0/18 (0.00)
Flatulence	21/22 (95.5)
Weight loss	23/25 (92.0)
Nausea	22/24 (91.7)
Abdominal cramps	19/24 (79.2)
Vomiting	10/19 (52.6)
Fever	9/21 (42.9)

^a^The denominators for symptoms vary because some respondents reported not being sure whether a symptom occurred. ^b^Diarrhea was defined as >3 bowel movements per day.

Frequencies, relative risks, and 95% confidence intervals were calculated for 12 main courses, 3 side dishes, 12 beverages, and 2 desserts; all items had been consumed by the 30 study participants. The only food item that showed a clear, statistically significant association with disease (relative risk [RR]=5.0 [1.4<RR<204], p=0.0045) was a salad side dish (side dish 1). All 25 persons who ate this salad became ill. The salad consisted of several sorts of vegetables (butterhead lettuce, mixed lettuce, red cabbage, white cabbage, carrots, cucumbers, and celery). Mixed lettuce (German: “Mixsalat”) was a designation for a commercially available mix of four varieties of green and red lettuce (lollo rosso, lollo bianco, oak leaf, and romaine lettuce). The other available salad (side dish 2) had been prepared with only two components: butterhead lettuce and mixed lettuce. Eight participants had chosen salad 2 and only one was not affected. Six of them had eaten salad 1 as well. Accordingly, one participant was affected who had only eaten salad 2. The three persons who did not eat any salad did not become ill. Therefore, the vehicle for the transmission of *Cyclospora* oocysts must have been one of the components common to both salad 1 and salad 2, i.e., one or more of the lettuce varieties or the fresh green leafy herbs (dill, chives, parsley, green onions) used to spice both salads. Chervil, which was also used for flavoring, had been stewed and can therefore be excluded. The pooled data for side dishes 1 and 2 (consumption of salad 1, salad 2, or both) showed a highly significant association with disease (p=0.0025; [1.4<RR<∞]). As none of the participants who had not eaten any of the two salads became ill, the exact RR risk for the either salad is not defined (denominator is zero). Neither of the two available dressings was associated with the disease. The hypothesis that all the main courses were uniformly contaminated, e.g., by the cook, was also tested, but no significant association was found. Only one participant had a history of traveling abroad in the 2 weeks before the luncheons.

The sources of implicated food items were traced through the analysis of invoices and delivery notes in the files of the restaurant owner and of retailers, wholesalers, and importers (for two lettuce batches, dill, parsley, and green onions) or by verbal report (chives).

The batches of butterhead lettuce and mixed lettuce went through four separate trading stages from the producer to an exporter/importer, a wholesaler, and a retailer before being bought by the restaurant proprietor. The butterhead lettuce batch had been grown in southern France, while the mixed lettuce batch had been grown in the province of Bari in southern Italy. Dill, parsley, and green onions were also grown in southern Italy (Naples, Eboli); only the chives had been grown in a greenhouse in Germany. Samples of the implicated batches were no longer available for microbiologic examination.

## Conclusions

To our knowledge, this foodborne outbreak of cyclosporiasis is the first reported from central Europe. Mesclun salad mix (also known as spring mix, field greens, or baby greens—a mixture of various types of baby lettuce leaves) and fresh basil have been involved in outbreaks in the United States (9,10). *Cyclospora* oocysts have also been isolated from lettuce in Peru (11), and Egypt (12) and from green leafy vegetables in Nepal (13). As *Cyclospora* must sporulate for at least 7 days at 25°C–30°C in the environment to become infectious (14), direct person-to-person or person-food-person transmission in a restaurant is nearly impossible. During the winter season in Germany, domestic sewage, surface water, and drinking water would not meet the temperature requirements for the sporulation process.

Therefore, the most probable routes of contamination of one or more of the implicated vegetables are fertilization with human waste or fecally contaminated water used to irrigate crops, prepare pesticides, or freshen or clean produce at their origin (15). In addition to contamination of field crops through the water route, we considered that seasonal field workers often do not have access to appropriate sanitary facilities.

The outbreak we described may represent a much larger problem. Public health offices and laboratories, general practitioners, and medical microbiology labs should be alerted to the fact that *Cyclospora* infections in central Europe can no longer be regarded as solely travel related. Physicians should be aware of the typical symptoms, the diagnostic methods, and the medical treatment of this emerging pathogen.
